# Potentially preventable hospitalizations—The ‘pre‐hospital syndrome’: Retrospective observations from the MonashWatch self‐reported health journey study in Victoria, Australia

**DOI:** 10.1111/jep.13460

**Published:** 2020-08-28

**Authors:** Carmel Martin, Narelle Hinkley, Keith Stockman, Donald Campbell

**Affiliations:** ^1^ Community Health, Monash Health Dandenong Victoria Australia; ^2^ Monash University Melbourne Victoria Australia; ^3^ MonashWatch and HealthLinks Chronic Care Community Health, Monash Health Dandenong Victoria Australia; ^4^ Staying Well Program Northern Health, Northern Hospital Epping Victoria Australia

**Keywords:** health transitions, patient journeys, post‐hospital syndrome, readmission, time series

## Abstract

**Rationale, Aims, and Objectives:**

HealthLinks: Chronic Care is a state‐wide public hospital initiative designed to improve care for cohorts at‐risk of potentially preventable hospitalizations at no extra cost. MonashWatch **(**MW) is an hospital outreach service designed to optimize admissions in an at‐risk cohort. Telehealth operators make regular phone calls (≥weekly) using the Patient Journey Record System (PaJR). PaJR generates flags based on patient self‐report, alerting to a risk of admission or emergency department attendance. ‘Total flags’ of global health represent concerns about self‐reported general health, medication, and wellness. ‘Red flags’ represent significant disease/symptoms concerns, likely to lead to hospitalization.

**Methods:**

A time series analysis of PaJR phone calls to MW patients with ≥1 acute non‐surgical admissions in a 20‐day time window (10 days pre‐admission and 10 days post‐discharge) between 23 December 2016 and 11 October 2017. Pettitt's hypothesis‐testing homogeneity measure was deployed to analyse Victorian Admitted Episode/Emergency Minimum Datasets and PaJR data.

**Findings:**

A MW cohort of 103 patients (mean age 74 ± 15 years; with 59% males) had 263 admissions was identified. Bed days ranged from <1 to 37.3 (mean 5.8 ± 5.8; median 4.1). The MW cohort had 7.6 calls on average in the 20‐day pre‐ and post‐hospital period. Most patients reported significantly increased flags ‘pre‐hospital’ admission: medication issues increased on day 7.0 to 8.5; total flags day 3, worse general health days 2.5 to 1.8; and red flags of disease symptoms increased on day 1. These flags persisted following discharge.

**Discussion/Conclusion:**

This study identified a ‘pre‐hospital syndrome’ similar to a post‐hospital phase aka the well‐documented ‘post‐hospital syndrome’. There is evidence of a 10‐day ‘pre‐hospital’ window for interventions to possibly prevent or shorten an acute admission in this MW cohort. Further validation in a larger diverse sample is needed.

## 1 INTRODUCTION

Potentially preventable hospitalizations are the subject of considerable concern for all‐patients, their families, hospitals, general practice and community services, and funders. In particular, many admissions of vulnerable and/or older adults may be avoidable. Of note many only provide ‘band‐aid’ solutions as they fail to address patients' multi‐dimensional care needs in the community. These admissions have uncertain impacts on the ongoing illness trajectory after hospital discharge,^1^, ^2^ and are associated with what has been described in the international literature as ‘post‐hospital syndrome’ and poor quality of health after discharge. What happens before admission? Does a post‐hospital syndrome exist or is it part of a continuum of poor health from before admission where non‐hospital interventions might be useful?

HealthLinks: Chronic Care (HLCC) is a funding‐neutral reform that aims to support Victoria's Public Health Services in adopting value‐based rather than activity‐based approaches that better identify patients at‐risk of hospitalization and respond to their care needs earlier. Monash Health is the largest hospital group in Victoria, Australia. It participated in the new funding model. Despite having very comprehensive community services attached to its hospitals and local communities, Monash Health had circa 4000 potentially preventable hospitalizations and implemented a HLCC funded service called MonashWatch (MW). The MW service started in a lower socio‐economic and ethnically diverse area of Melbourne proximate to the Dandenong Hospital. The MW service pilot commenced on 23 December 2016 with a telehealth component called Patient Journey Record System (PaJR) supporting regular structured phone calls to patients incorporated into a coaching and anticipatory care model.

This article presents a retrospective analysis of phone calls to patients who had at least one acute non‐surgical (ANS) admission in the first 10 months of the MW service in order to identify potential patterns before ANS admission and after discharge. The objective is to identify significant changes in call patterns detectable before admission and after discharge.

### 1.1 Theory

Post‐hospital syndrome is an internationally recognized phenomenon after hospital discharge that has been defined as ‘a transient period of generalized susceptibility to disease as well as an elevated risk for adverse events, including hospital readmission and death’.^3^ Theories of causation focus on decreased physiological and emotional resilience^2^ acquired during an admission which do not (fully) compensate for the illness for which the patient was originally admitted. Stresses on vulnerable or frail people can complicate hospitalization and persist after discharge and include medication and treatment impacts and psychosocial decompensation. In the longer term, most older patients with multi‐morbid conditions will have an irreversible loss of systemic resilience and a fast(er) decline following hospital admissions, particularly if they are in the last years of life, admitted to the ICU, or being from a particularly chronic disease group such as heart and respiratory failure or dementia.^4^, ^5^, ^6^ There has been a presumption that disease self‐management breaks down, on one hand, and that frailty including dementia trajectories decline more rapidly following admissions and discharge, on the other hand.

Much effort to address potentially preventable hospitalizations has focused on post‐hospital transitions of care.^7^ International literature indicates that transitional care interventions can successfully support older patients with complex conditions^3^ to reduce readmissions.

Little attention has been paid to what leads to an acute ANS hospital admission in adults and older people with frequent admissions.

### 1.2 The service

The HLCC program employs analytics on hospital data to identify patients predicted to be at‐risk of ≥3 acute hospitalizations in the subsequent 12 months^8^ and incentivizes hospital systems to improve potentially preventable hospitalization admissions within cost containment.

The HLCC algorithm identifies an eligible cohort of patients with service parameters including recent acute admissions and emergency department visits. Patient parameters include age, residence status, smoking, and chronic conditions—gastrointestinal disorders, renal disease, asthma, chronic obstructive pulmonary disease, rheumatoid arthritis, diabetes, pancreatic conditions, cirrhosis/alcoholic hepatitis, excluding serious mental and psychotic illnesses, dialysis and cancer treatments because there are other initiatives for these groups. The Victorian Department of Health provides updated ‘HLCC eligible cohort’ lists to hospital groups and funds care improvement initiatives based on projected reductions in admission costs.

Previously, MW has demonstrated that resilience theories can provide a comprehensive operational framework^5^ to address admissions. PaJR addresses the risk of potentially preventable hospitalizations^7^ in a longitudinal trajectory. It was initially developed and validated with an Irish primary care cohort, before being deployed by Monash Health.

**FIGURE 1:**
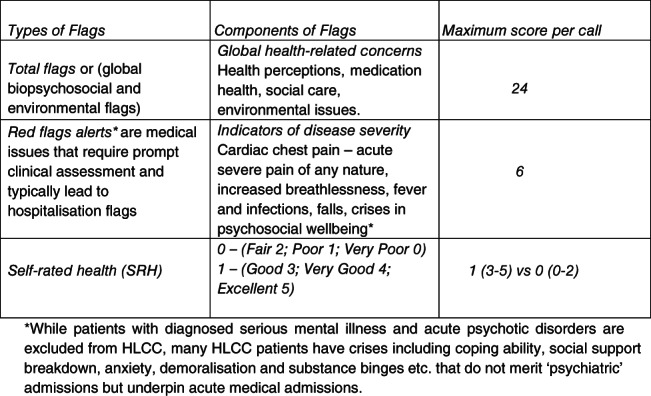
Alerts (Flags) generated by the Patient Journey Record System (PaJR) in MonashWatch Service

The MW service monitored the participating HLCC cohort through outbound phone calls by Telecare Guides trained call operators using PaJR data system. Outbound phone calls is a term used to describe the process of regular outreach through phone calls by Telecare Guides, rather than waiting for the patients to call in if they perceive a problem. Patients, caregivers, or professionals can call‐in between scheduled calls (inbound calls), as required. The PaJR system collects data from all the semi‐structured calls related to all patients enrolled into the program. Data analysis of self‐reported observations of daily health and living generates flags enabling the proactive management of MW patients (see Figure 1 for a detailed list of PaJR flags analysed in this article). Triaging and analysing these flags may uncover health deterioration, medication concerns and lack of support—often the root causes behind a person's decline.^9^ More alerts per a call indicate vulnerability to worsening health and the potential for hospital admission.^10^

## 2 METHODS

The study selected a 20‐day *time window* of a period of 10 days before an ANS admission and 10 days after discharge, as amenable to investigation. A retrospective descriptive time series analysis was conducted on patients allocated to the MW intervention cohort of a pragmatic clinical trial, who had long enough participation to have received ≥44 calls. In this analysis, the admission period is collapsed to day 0 (zero) such that day −10 to −1 represent days before admission and day 1 to 10 represent days after discharge.

*Total flags* (global biopsychosocial concerns flags) and *red flags* (disease symptoms of concern flags) and self‐rated health assessments were extracted from the PaJR system. This data were collected during phone calls between the MW staff and patients and integrated with patients' admission data from the Victorian Admitted Episodes Dataset/Victorian Emergency Minimum Dataset. In Victoria, an acute or emergency admission occurs when a patient is admitted to an emergency department short stay ward or an inpatient ward, irrespective of the origin of their journey—the emergency department, outpatients, or direct General Practitioner (GP) admission.

XLSTAT is a statistical package^11^ from which Pettitt's non‐parametric test was selected to test for a shift in the central tendency (or homogeneity) of the time series of alerts per call. It is a test suitable for all continuous distributions for detecting, in a data series, if there is a time at which a distinct change occurs, that is, a transition or tipping point, using hypothesis testing.^11^

For Pettitt test, XLSTAT provides a *K* value and *P* values using Monte Carlo resampling and uses two‐sided alpha ≤ .05 standard, that is, there is less that a 5% chance that an hypothesis is rejected due to chance.^11^ Alpha or *α*, is the probability of rejecting the null hypothesis when it is true. A significant *P* value at alpha ≤ 0.05 rejects H0‐hypothesis (that there is no statistically significant change in flags) and accepts the HA‐hypothesis (that there is a statistically significant change at a particular time point).

Monash Health's Health Research Ethics Committee (HREC) provided ethics approval for the MW pilot service and its internal evaluation by the MonashWatch team.

## 3 FINDINGS

The study describes the general characteristics of a MW cohort of 103 patients with an age range 65 to 100 years (mean 71 ± 15 SD; median 74 years with 59% being male in the period 23 December 2016‐11 October 2017). It demonstrates a ‘pre‐hospital syndrome’ based on changes in flags and their sub‐components in the time series 10 days before admission. These changes persisted for another 5 to 10 days post‐hospital discharge.

### 3.1 General characteristics

The MW cohort, collectively, had 263 ANS admissions and 768 PaJR calls in the 20‐day *time window* (10 days before and 10 days after hospitalization) with a median of 7.6 calls per person; and in total 5583 calls with a median of 5.5 calls per participant per month. The 20‐day window PaJR calls were distributed evenly in relation to admission and discharge. Day 0 represents the admission period irrespective of length of stay (LOS). Calls were not intentionally made during admissions, although about six calls were made to people as the Telecare Guides were unaware that they were in hospital.

LOS of the ANS admissions in this cohort ranged from <1 to 37.3 bed days with a skewed distribution (mean 5.8 ± 5.7 SD; median 4.1 days). Emergency department short stay admissions were coded as 1 day if kept overnight.

**FIGURE 2:**
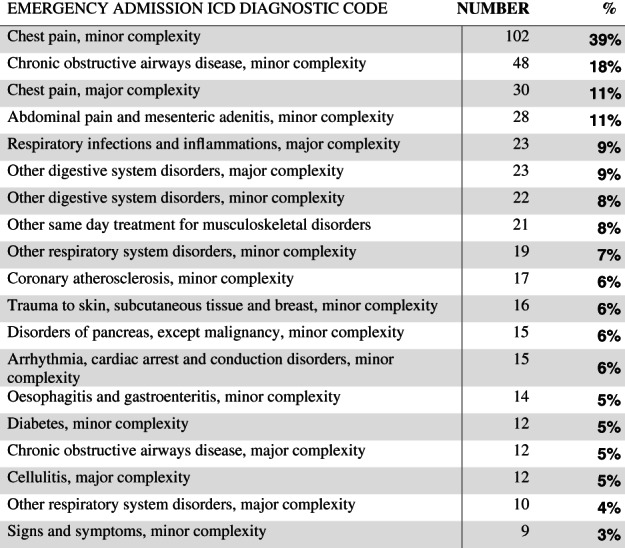
The most common International Classification of Diseases (ICD) diagnostic codes for emergency (acute nonsurgical) admissions in MonashWatch. Complexity is defined according to the Australian Refined Diagnostic Related Groups (AR‐DRG) classification process based on multiple factors including cost, clinical comorbidities, and length of stay^10^

The top six reasons for admission were: chest pain, minor complexity; chronic obstructive airways disease, minor complexity; chest pain, major complexity; abdominal pain and mesenteric adenitis, minor complexity; and respiratory infections and inflammations, major complexity (Figure 2).

### 3.2 Flag patterns

The MW cohort flag patterns in the 20‐day time window differed from their (the same MW cohort) other calls:

- *Total flags* per call in the *time window* averaged 3.0 ± 1 and median 2 flags per call vs all calls by these same patients outside of the admission window under study with 1.0 ± 1 and median 0 flags per call, respectively.
- *R* *ed flags* averaged 2 ± 1 per call with median 1 inside the study window compared with an average 1 ± 1 and median 0 flags per call of these same patients outside of the admission window.
- Self‐Rated Health in these same patients was fair to good before entry to window and median (very poor to fair) inside the study window until day 5 post‐hospital vs (good to excellent) in other calls.

#### 3.2.1 Time series characteristics

**FIGURE 3:**
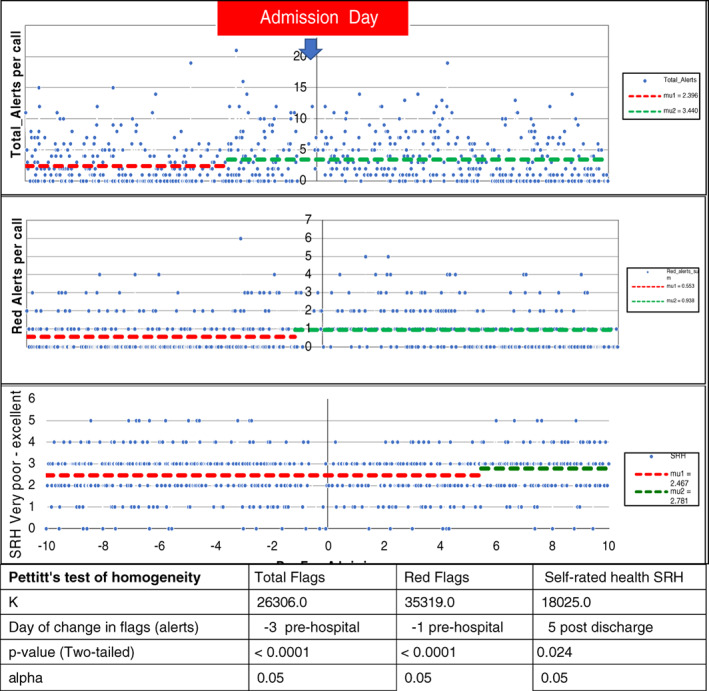
Total and Red flags (also called alerts) time series (with day 0 representing the admission period irrespective of length or stay) demonstrate a statistically significant shift before the day of admission day 0, at day −3, and day −1, respectively. Self‐Rated Health demonstrates a stable pattern of fair‐good from entry an improvement on day 5 post‐discharge to good. This is based on 768 calls and 103 patients and 263 who were admitted as an emergency admission. The significant *P* value indicates that the shift is a statistically significant shift using Pettitt's non‐parametric test of homogeneity

The time series of *total flags* and *red flags* within the 20‐day window of an ANS hospitalization revealed a statistically significant shift towards higher levels of *total* and *red flags* before admission that there were no detectable changes. The plots of flags demonstrate the statistically significant shifts in homogeneity for Total flags, Red flags and Self‐Rated Health but with the considerable variation which is discussed below (see Figure 3).

#### 3.2.2 Transitions in flags before ANS admissions

**FIGURE 4:**
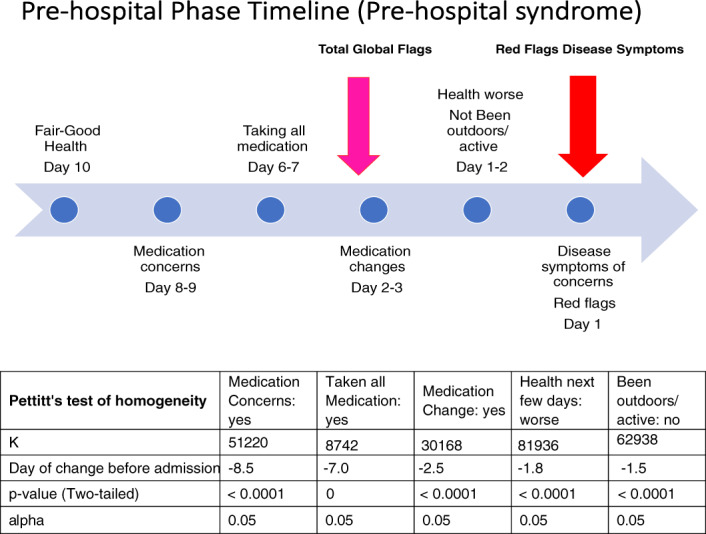
Pre‐hospital phase. Specific alerts in time series demonstrate a statistically significant shift in relation to admission—on day 0. This is based on 768 calls and 103 patients and 263 acute nonsurgical emergency admission. The significant *P* value indicates that the shift is a statistically significant using Pettitt's test of homogeneity

Medication use, self‐reported health, pain, feeling depressed, not coping, concerns about caregiver and serious symptoms were investigated in the 10‐day timeline before admission (see Figure 4):

- Medication issues
  - Patient‐reported medication concerns statistically increased at 8.5 days (*P* < .0001).
  - Reporting ‘having taken all their medication’ significantly increased at −7.0 days (*P* = 0). Patients were specifically asked this question if they had previously indicated medication concerns. This indicates that people remained compliant even if they had concerns.
  - Seventy‐five percent of patients reported medication changes during the 10‐day window before and after hospitalization. This increased to 82% of calls on day −2.5 before an admission (*P* < .0001). This generally involved contact with their GP.
- Self‐reported health transitions
  - Health perceptions that ‘the next few days might be worse, very much worse or maybe worse’ significantly increased at day −1.8 (*P* < .0001).
  - ‘Been outdoors or walked around for 20 to 30 minutes in past 24 hours’ significantly declined at day −1.5 (*P* < .0001).

A pre‐hospital *timeline to admission* was constructed incorporating the statistically significant Total and Red flags and selected sub‐components. A sequence of fair‐poor health, medication concerns and compliance, medication changes, general health decline was followed by a significant increase in disease symptoms before the ‘tip’ into admission (see diagram in Figure 4).

There was not a unique shift in the time series of other alert components. For example, reported levels of pain did not statistically shift in homogeneity (*P* = .12) in the *time window*. While Self‐Rated Health, feeling depressed, not coping and caregiver concerns time series did not shift before admission, they demonstrated a positive shift (improvement) 5 days after discharge (*P* < .0001)—not shown in the diagram.

### 3.3 Non‐alerters

The majority of patients (83%) demonstrated this pre‐hospital phase. They reported 4.6 total alerts per call and 1.6 red flags per call in the pre‐hospital time window, with chronic condition exacerbations. While most patients had red flags and alerts prior to hospitalization, 17% patients did not alert at all in the 3 days before admission. The most common admission diagnosis was chest pain of minor complexity which were <1 bed days in the Emergency Department (ED) short stay (deemed an admission by DHHS). All were from cultural minorities and non‐English speaking backgrounds (see Box 1)

**Box 1 Profiles of patients who did not ‘alert’ before an acute admission**

- Patient X was the most frequent admitter—a middle‐aged male, living alone who had 27 admissions for chest pain, minor complexity of <1 day and denied any problems before and following admission and most pertinently, in the 3 days before admission.

The other non‐alerting patients had multimorbidity and more varied admission patterns.

- Patients A was a 75‐year‐old lady living alone, who had five admissions with zero *total* and *red flags* in the prior 3 days. Admissions were for abdominal pain and mesenteric adenitis, minor complexity, oesophagitis and gastroenteritis, minor complexity, hypertension, minor complexity, kidney and urinary tract infections, major complexity, and respiratory infections and inflammations, major complexity.
- Patients (B, etc) who reported zero *flags* and *red flags* 3 days before admission, all lived alone and were males. Of this sub‐group, admissions were predominantly for minor complexity conditions including chest pain, other digestive systems disorders, same day treatment for musculoskeletal disorders, and trauma.

## 4 DISCUSSION

The MonashWatch pilot service study indicates that 83% of the ANS admissions were preceded by a prodromal phase or a ‘pre‐hospital syndrome’. Medication issues and health perceptions potentially amenable to early interventions by a GP or other practitioner were identified from alerts. This pre‐hospital phase is characterized by a sequence of increase in self‐reported experiences of medication issues, health‐related disturbances, and significant concerns about disease symptoms before an admission. These findings challenge the notion of their being a unique post hospital syndrome but demonstrate a continuum of poor self‐assessed health from before to after admission.

### 4.1 A pre‐hospital syndrome

A pre‐hospital phase was identified at the beginning of the 10‐day observation window in the majority of patients. A ‘cascade’ of transitions with increased alerts preceded admission. There were statistically significant shifts to increased: medication concerns about day −9 to −8; medication adherence around day −8 to −7; and medication changes at days −3 to −2. A shift to increased total global alerts were reported on day −3, with decreased activity and increased anticipated worse or uncertain health reported between day −2 to −1. There was a shift to increased serious symptoms of concern around 1 day before admission. Patients who did not report PreHS transitions fell into 2 categories—serial admitters for chest pain and minor conditions, and patients living alone with multiple morbidities, all from non‐English‐speaking backgrounds. This subset who did not alert may not have observed any changes or experienced cultural barriers to communicating their experiences. This warrants further investigation.

The pre‐hospital phase appears continuous with the post‐hospital syndrome,^3^ despite the intervening hospitalization in the MW cohort. While the post‐hospital care transitions literature^1^, ^2^, ^3^ demonstrates short‐term health improvements with interventions, the trajectories of community dwelling older patients with frailty demonstrate that admissions and emergency department use^9^ are followed by significant decline in health and survival over time.^4^, ^5^ Each admission of an older frailer multi‐morbid patient is likely to result in less resilience—the ability to bounce back to a pre‐prodromal state—and ongoing decline.^5^ Although the MW cohort is identified by predicted frequency of admissions, frailty at baseline was the best predictor of an acute hospital admission^12^ indicating the applicability of other longitudinal studies. Thus, it may be important to avert unnecessary hospitalizations, even short ED admissions or visits to improve survival trajectories, as well as for cost implications.^13^

### 4.2 Tipping points and transitions

The patterns of alerts demonstrate a series of worsening alerts, flagging medication, and wellbeing concerns before a significant tipping point into an acute admission. A tipping point is the point at which a series of small changes or incidents becomes significant enough to cause a larger, more dramatic change.^14^ Tipping points or the prediction of potential tipping points is an important component of clinical care^14^; however, the prediction is very short term in the non‐linear dynamics of illness trajectories^15^ and transitions occur within a few days.

### 4.3 Implications of the pre‐hospital syndrome

On a practical note, medication changes were very frequent before and after admission. In the pre‐hospital phase, contact with GPs and other professions around medication concerns and changes may be an alarm sign and provide an opportunity for early intervention. Contacts related to medication management, offer an opportunity to investigate whether these presentations are actually medication and treatment‐related or proxies for worsening illness and/or psychosocial and environmental challenges.

It is difficult to disentangle the impact of the internal systemic stress triggered by an emerging acute illness from external stressors related to medical treatment, health system, psychosocial and/or environmental issues; and the last year of life trajectories do not follow a predictable pattern based on the condition leading to death.^13^ Indeed, many such hospitalizations may signify an unavoidable decline—reflecting the dynamic shifts between health and disease^15^ that hospital admission cannot prevent and/or revert.

Regardless, these observations demand a much more integrated approaches to post‐hospital transitional care aimed at ameliorating the ongoing unavoidable decline. Our telehealth model approach aims to managing a high‐risk patient group with unstable trajectories across a pre‐ and post‐admission 10‐day *time window*. Monitoring concerns the identification of tipping points—emerging amongst a wide range of health, psychosocial and environmental features. Whether biometric monitoring in general or conversational self‐report monitoring during this phase on the illness trajectory is needed for improved clinical care remains an unanswered question.

### 4.4 Limitations

Efforts to understand health transitions are beginning to become evident in the health care and frailty literature,^16^, ^17^ but without consensus as how to define a tipping point, particularly in an irregular time series in the real world nature of telehealth calls, remains challenging. The sequence of events cascading to an acute hospitalization has received little attention in the literature, preventing a contextual comparison with other literature. The sample size is small. There is a need for further research in a larger samples and other cohorts identifying potential tipping points and phases to validate these findings. How best to address such trajectories with a window of only a few days also requires more investigation.

## 5 CONCLUSION

This study of pre‐hospital and post‐hospital admission trajectories in a cohort of high‐risk individuals identified a ‘pre‐hospital syndrome’ characterized by a series of tipping points in medication and Self‐Rated Health complaints that result in acute hospitalization. Such tipping points have a clinical as well as a statistical meaning in that medication concerns and changes prefigure worsening health and significant disease symptom concerns. Practitioners can be alerted to reports of ‘medication not working’ and ‘making medication changes’ with a general worsening of health perceptions which may precede serious symptoms that tip a patient to hospitalization. Telehealth with phone calls has demonstrated these patterns and interventions in one cohort.

## CONFLICT OF INTEREST

Carmel Martin is a co‐developer of the PaJR software and a health services research adviser to PHC Research Pty Ltd which owns the PaJR software. Keith Stockman, Narelle Hinkley and Donald Campbell declare no conflict of interest.
